# Longitudinal Analysis of Prevalence and Risk Factors of Rifampicin-Resistant Tuberculosis in Zhejiang, China

**DOI:** 10.1155/2020/3159482

**Published:** 2020-02-12

**Authors:** Zhengwei Liu, Mingwu Zhang, Jianmei Wang, Songhua Chen, Beibei Wu, Lin Zhou, Aizhen Pan, Weibing Wang, Xiaomeng Wang

**Affiliations:** ^1^The Institute of TB Control, Zhejiang Provincial Center for Disease Control and Prevention, Zhejiang, China; ^2^Department of Epidemiology, Fudan University, Shanghai, China

## Abstract

**Objectives:**

To investigate the factors associated with rifampicin-resistant tuberculosis among drug resistant tuberculosis patients and to determine the correlation of rifampicin-resistant TB with MDR-TB in a high MDR-TB burden province of china.

**Methods:**

A retrospective longitudinal analysis on four surveys of anti-TB drug resistance done in 1998, 2003, 2008, and 2013 in Zhejiang province, China. 4289 sputum-smear microscopy positive suspected tuberculosis patients were eligible at 30 investigation points, chosen by stratified random sampling at survey sites from all over the province. Culturing samples in L-J medium and the drug-susceptibility testing for the 4 first-line anti-TB drugs were performed to all patients. Multivariate logistic regression was carried out to determine the factors associated with the rifampicin-resistance in the study population.

**Results:**

Overall, there were 3832 patients with positive mycobacterial cultures, and 2813 of the isolates (73.4%) were susceptible to all 4 first-line drugs. Analysis of rifampin monoresistant (RMR) TB indicated the prevalence was 1.1% in new cases and 3.4% in previously treated cases. Among the 359 rifampicin resistant TB (RR-TB) cases, 279 (77.7%) were also resistant to isoniazid, indicating MDR-TB. From 1998 to 2013, the proportion of MDR-TB among rifampicin-resistant TB cases varied between 80.0% and 87.5% (P for trend: 0.768) among previously treated cases and varied from 68.6% to 79.5% (P for trend: 0.403) among new cases. Among previously treated patients, those who received treatment for less than 6 months were less likely to have drug resistant TB (OR: 0.40, 95% CI: 0.16–0.97) or MDR-TB (OR: 0.24, 95% CI: 0.07–0.81). Patients who received anti-TB treatment in a general hospital were less likely to develop MDR-TB than those treated in a TB clinic (OR: 0.08, 95% CI: 0.01–0.72).

**Conclusion:**

This study highlights a high proportion of RMR-TB among new RR-TB cases in Zhejiang, China. The management of treatment with rapid and accurate diagnosis of MDR-TB other than only relying on RIF susceptibility testing is crucial for improving adherence and outcomes in patients with drug-resistant TB.

## 1. Introduction

Drug-resistant tuberculosis (TB), including rifampicin (RIF)-resistant TB (RR-TB), multidrug-resistant (MDR) TB (with resistance to at least isoniazid and rifampicin), and extensively drug-resistant (XDR) TB (with resistant to rifampin, isoniazid, any fluoroquinolone, and at least one of three injectable second-line drugs) are major threats to the control of TB worldwide. An estimated 3.4% of new TB cases and 18% of previously treated TB cases had MDR/RR-TB in 2018 globally. While in China, 7.1% of new cases and 24% of previously treated cases had MDR-TB, higher than the global averages [1]. Thus, strength MDR-TB management is crucial to control the prevalence of MDR-TB in China.

Rifampicin and isoniazid are key anti-TB drugs that are used for initial treatment and for the retreatment of TB patients. The availability of rifampin allows successful treatment in cases with organisms resistant to isoniazid, streptomycin, or other agents. However, when resistance to rifampin occurs in *Mycobacterium tuberculosis*, more than 90% of cases have been associated with isoniazid resistance [2], which has resulted in the prospects for successful treatment are greatly diminished. Patients who were infected by *Mycobacterium tuberculosis* with rifampicin or isoniazid resistance require more intensive treatment regimens [3].

In order to strengthen the management of rifampicin resistance, rifampicin-resistant TB (RR-TB) was previously defined as resistance to rifampicin with or without resistance to other anti-TB drugs by the WHO. Thus, this category includes RMR-TB (rifampicin monoresistant TB), RPR-TB (rifampicin polydrug resistant TB), MDR-TB, and XDR-TB [4]. As a new strategy for the MDR-TB control program, the WHO recommended to use rifampicin resistance as a proxy for MDR-TB and detected and treated rifampicin-resistant TB patients in 2016 [1]. More retrospective analysis including worldwide research, however, showed that the proportion of rifampicin-resistant isolates that were isoniazid-susceptible (based on phenotypic drug-susceptibility testing) varied widely [5–7].

Zhejiang province is one of the most developed provinces in China and has a high burden of multidrug-resistant TB [8, 9]. For ensuring effective management of the development of tuberculosis drug resistance, Zhejiang province has constructed routine surveillance of anti-TB drug resistance for monitoring the effectiveness of TB control programs since 1998. Though we analyze the trend of the drug resistance, especially monitoring the development of MDR-TB and XDR-TB, no systematic studies determined the associated risk factors with rifampicin resistance in this population, and there is still more unknown about longitudinal trends in rifampicin resistance in this region. In this study, we constructed a retrospective analysis on anti-TB drug resistance in the population of Zhejiang from 1998 to 2013 to determine the longitudinal changes of rifampicin-resistant TB, and to determine the correlation of rifampicin-resistant TB with MDR-TB.

## 2. Materials and Methods

### 2.1. Study Participants

This study was a retrospective analysis of a sequence survey data of drug resistance in TB. In 1998, according to the recommendation of WHO'S guidelines for surveillance of drug resistance in tuberculosis, the first survey was conducted by Zhejiang Provincial Center for Disease Control and Prevention in Zhejiang province. A cluster-randomized sampling was obtained to obtain a representative sample of patients with TB according to previous description [10]. Based on the prevalence of rifampicin resistance and the notification number of new smear-positive patients across the entire province, 30 counties were selected. At each site, 30 new smear-positive patients were enrolled and all previously treated smear-positive patients were enrolled. The same protocol was used in each of the 4 rounds of this survey in 1998, 2003, 2008, and 2013, respectively, to determine changes of MDR-TB over time.

All patients had registered smear-positive TB during the survey period (1998–2013) at TB clinics selected as sample sites and were new cases or previously treated cases. New cases were those who never received TB drugs or received treatment for less than 1 month. Previously treated cases were those who received previous TB treatment for 1 month or longer. All patients were active TB cases with bacteriological confirmation by sputum cultured with Löwenstein-Jensen (L-J) medium. Newly diagnosed patients provided 3 sputum specimens (spot, morning, and night) and previously treated patients provided 2 sputum specimens (spot and morning or night). Extrapulmonary TB patients were excluded from this study.

### 2.2. Data Collection

Two trained interviewers separately interviewed each patient with a standard questionnaire to collect sociodemographic data and information on previous treatments for TB, but more detailed sociodemographic information was only available in 2013. No information on human immunodeficiency virus (HIV) status was collected. Discrepant interview data were resolved by a third interviewer via telephone or visitation.

### 2.3. Bacteriological Testing

At least 2 sputum samples were obtained from each patient before initiation of treatment. These samples were tested by smear microscopy and by culturing in solid Löwenstein-Jensen (LJ) medium in county-based TB laboratories. For culture isolation, each specimen was treated with 1 : 1 volume of 4% NaOH to sputum specimen and then homogenized by vigorous stirring. A 0.1 mL aliquot of the resulting sample was inoculated into 2 tubes of acidified LJ medium and incubated at 37°C. The culture was assessed during week-1 for rapidly growing bacteria and every week thereafter for slower growing bacteria; if no bacteria appeared by week-8, the result was recorded as negative. Cultures with growing colonies were sent to the Zhejiang Provincial TB Reference Laboratory (PTRL) for identification and drug-susceptibility testing. Growth characteristics, colony morphology, and inhibition by p-nitrobenzoic acid were used to differentiate *Mycobacterium tuberculosis* from other mycobacteria.

Indirect drug susceptibility of the culture-positive isolates was detected by the proportion. After subculturing in L-J medium, indirect drug susceptibility was determined by the proportion method, with the following concentrations for the 4 first-line anti-TB drugs: 0.2 *μ*g/mL for isoniazid, 4 *μ*g/mL for streptomycin, 40 *μ*g/mL for rifampicin, and 2 *μ*g/mL for ethambutol. The strain was declared resistant to the corresponding drug when the growth rate was higher than 1% compared to the control. [11].

### 2.4. Quality Control for DST

The laboratory had participated in the external quality control programs by the Republic of Korea Supranational Reference Laboratory and the Chinese National TB Reference Laboratory. The standard reference strain H37Rv (provided by the Chinese National TB Reference Laboratory) was used for DST internal quality assurance.

### 2.5. Statistical Analysis

Two individuals independently entered data into SPSS statistical software, version 16.0 (SPSS Inc., Chicago, USA). The drug resistance trend over time was analyzed using the Cochran–Armitage test for trend. The factors associated with drug-resistant TB were examined by a univariate and multivariate logistic regression model. The odds ratio (OR), 95% confidence intervals (CIs), and *P*-values were used to evaluate factors associated with MDR-TB and drug-resistant (but not MDR) TB with a significant level at 0.05.

### 2.6. Ethics Statement

The Ethics Committee of Zhejiang Provincial Center for Disease Control and Prevention provided ethical approval and all participants provided written informed consent.

## 3. Results

### 3.1. Characteristics of the Patients

**Figure 1:**
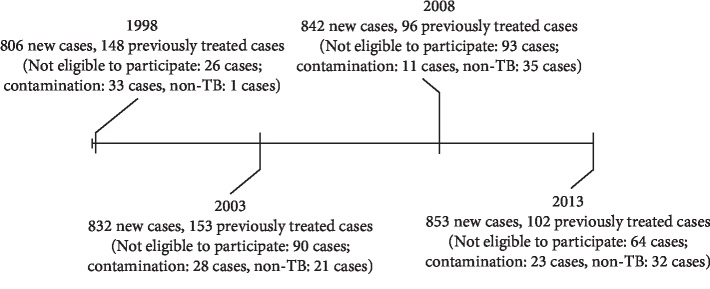
Enrollment of tuberculosis patients during the 4 surveys (1998–2013).

The sequence surveillances patient recruitment began on April 1, 1998, and ended on December 31, 2013 (Figure 1). We initially enrolled 4289 patients. Specimens from 361 patients (8.42%) did not grow mycobacteria or were contaminated, and specimens from the other 3928 patients (91.6%) yielded positive mycobacterial cultures, with 96 cultures (2.44%) identified as non-TB mycobacteria. DST results were available for 3832 patients (89.3% of 4289 patients), 3333 new cases and 499 previously treated cases.

### 3.2. Prevalence of MDR-TB and Rifampicin-Resistant TB

A total of 2814 isolates from these 3832 patients (73.4%) were susceptible to all 4 first-line anti-TB drugs (isoniazid, rifampin, ethambutol, and streptomycin). Our analysis indicated that 22.7% of new cases (95% confidence interval [CI]: 21.3–24.1%) and 52.7% of previously treated cases (95% CI: 48.3-57.1%) were resistant to at least 1 of the first-line drugs (Table 1).

Overall, 359 isolates were RIF-resistant (resistant to at least RIF), and 279 of these isolates were from MDR-TB patients. The percentage of RR-TB among new cases was 5.4% and that among previously treated cases was 36.1%. The percentage of MDR-TB in new cases (3.8%) was lower than in previously treated cases (30.3%). The prevalence of RMR-TB was 1.1% among new cases and 3.4% among previously treated cases. The prevalence of RMR ranged from 17.1% to 25.5% in the 179 new RR-TB cases, and from 6.3% to 12.3% in the 180 previously treated cases (Table 2). This difference was significant (*χ*^2^ = 9.126, *P* < 0.01). However, there were no significant differences for males and females and no significant differences among the 4 years of testing. On the other hand, the proportions of INH resistant TB patients were 7.0% and 9.2%, respectively, in new and previously treated RIF-susceptible TB cases.

Table 2 also showed that 4.3% of new patients and 5.9% of retreatment patients have resistance to isoniazid (monodrug resistance), whereas it was 0.9% and 2.9% for rifampicin monodrug resistance.

### 3.3. MDR-TB Prevalence in Rifampicin-Resistant TB Patients

**Figure 2:**
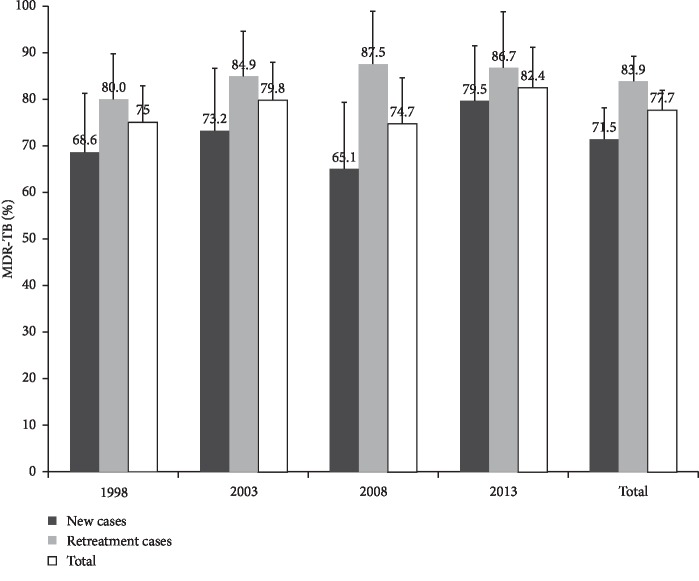
Percentage of MDR-TB cases among rifampicin-resistant TB cases, from 1998 to 2013.

Among the 359 RR-TB cases, 279 (77.7%) were also resistant to isoniazid, and therefore classified as MDR-TB. Thus, the overall probability that a subject who was positive for RR-TB had MDR-TB was 71.5% (128/179, 95% CI: 63.3–77.0) among new cases and 83.9% (151/180, 95% CI: 77.8–88.8) among previously treated cases. From 1998 to 2013, the proportion of MDR-TB varied between 80.0% and 87.5% (P for trend: 0.768) among previously treated cases, and varied from 68.6% to 79.5% (P for trend: 0.403) among new cases (Figure 2).

### 3.4. Risk Factors for DR-TB and MDR-TB

Table 3 shows the results of the multivariable analysis of factors associated with drug-resistant TB in new cases and previously treated cases in 2013. Among new cases, none of the analyzed factors were significantly associated with drug resistant TB or MDR-TB. However, among previously treated cases, those who received treatment for less than 6 months were less likely to have drug resistant TB (OR: 0.40; 95% CI: 0.16–0.97) or MDR-TB (OR: 0.24; 95% CI: 0.07–0.81) than those who received treatment for 6 months or more. In addition, among previously treated cases those who received anti-TB treatment in designated general hospitals were less likely to have MDR-TB than those treated in TB dispensaries (OR: 0.08; 95% CI: 0.01–0.72).

## 4. Discussion

The increasing incidence of MDR-TB is a major concern for TB control in a high burden country. Strengthening the management of MDR-TB program especially in early diagnosis of MDR-TB is crucial to reduce the risk of MDR-TB transmission. Another study shows that 59% of the MDR-TB patients were from new TB cases in Zhejiang province [8]; screening for MDR-TB among the new TB patients is crucial. The results of this study further confirm the high prevalence of drug-resistant TB in Zhejiang Province, although the prevalence decreased slightly during the 15-year study period. In 2013, 22.5% of new TB cases and 47.1% of previously treated TB cases had drug-resistant TB. At that time, 4.1% in new cases and one-quarter of patients with previously treated TB had MDR-TB, on par with the averages of national survey of drug-resistance in tuberculosis (5.7% and 25.6%) [12]. Although this province has a serious epidemic of drug-resistant TB, the detection rate for MDR-TB is still very low in China. The results of the 2013 survey showed that 5.2% of new cases and 8.8% of previously treated cases had TB that was resistant to either isoniazid or rifampicin, meaning that these individuals are more likely to develop MDR-TB.

The WHO report on drug resistant TB indicated that mono- and polyresistant TB are more common than MDR-TB [1]. An abundance of monoresistant cases can lead to the spread of resistance, and eventually to MDR, if patients do not receive proper treatments. Furthermore, treatment of mono- and polyresistant TB using the WHO standardized first-line drug regimens increases the risk of treatment failure and can even lead to amplification (acquisition of additional resistance) and MDR-TB [13]. Very few randomized clinical trials have assessed the best treatment for mono- or polyresistant TB [14]. Thus, current practice guidelines are based on limited data from observational cohort studies and the recommendations of expert panels.

Accurate diagnosis of MDR-TB is needed to successfully treat TB and to reduce transmission [15, 16]. According to the WHO report, only 16% of MDR-TB patients received correct diagnoses and timely treatment [1, 17]. Delayed laboratory diagnosis is a major reason for treatment delay because the traditional phenotypic DST takes at least 2 to 3 months. Among the methods recently introduced for the rapid diagnosis of MDR-TB, Xpert MTB/RIF is the most commonly used because it is easy to use, sensitive, and specific [18]. Previous studies showed that drug resistance correlates with certain gene mutations in TB bacilli. In particular, more than 96% of *M. tuberculosis* strains with various levels of rifampicin resistance have mutations in the gene targeted by rifampicin (*rpoB*) [19, 20], and these mutations occur in a 27 amino acid region [21]. Some studies estimated that 85–90% of RR-TB are also resistant to isoniazid [22, 23], particularly in geographic areas with high prevalence of MDR-TB [24]. Therefore, many TB control programs throughout the world use rifampicin resistance as an indicator of MDR-TB.

The WHO report on TB showed that 160,684 patients were enrolled in MDR-TB treatment programs, and 82% of them had confirmed MDR-TB and 21,570 had RR-TB (determined by Xpert MTB/RIF) [1]. China, with support of the China Global Fund Multi-drug Resistant Tuberculosis project, adopted Xpert MTB/RIF as a technology for rapid diagnosis in county-level TB laboratories. However, some studies, including a systematic review, showed that the rapid test for molecular rifampicin resistance alone does not accurately predict phenotypic MDR-TB in areas with low prevalence of rifampicin resistance [25]. For example, a recent review of the Xpert MTB/RIF assay reported high rates of false-positive rifampicin resistance in a hypothetical cohort of 1000 individuals suspected of having MDR-TB, corresponding to a population prevalence rate of 2% MDR-TB [26]. Our results also indicate that rifampicin-resistant isolates are not necessarily resistant to isoniazid in a setting with a high prevalence of RR-TB. The cooccurrence of decreased isoniazid monoresistance and increased rifampicin resistance shows that rifampicin resistance does not predict MDR-TB in many patients and thus emphasizes the importance of a confirmatory DST. Although *rpoB* mutation-based testing may be effective for measuring rifampicin resistance and MDR-TB, false-positive results occur in some cases, in which smear-positive TB is rifampicin-resistant via GeneXpert MTB/RIF but phenotypically susceptible to rifampicin [27].

Although rifampicin resistance can be useful as a surrogate for screening in many settings [16], several recent papers concluded that rifampicin resistance is an unreliable indicator of MDR-TB in populations with low prevalence of MDR- TB [28, 29]. When considering a positive rapid rifampicin resistance result in a TB patient, results from a conventional DST, using solid or liquid culture, will help determine the best treatment. Thus, while awaiting DST results in a patient with a high risk of MDR-TB, the use of an MDR-TB regimen with isoniazid may be appropriate if resources permit. However, further studies are needed to assess the costs and benefits of this strategy.

The current strategy of using the rapid RR-TB diagnosis method to detect MDR-TB or pre-MDR-TB, may increase the risk for overdiagnosis and overtreatment. Previous research reported that patients with RMR may have an increased risk of poor treatment outcomes, even in settings with a low HIV burden and after adjusting for HIV status [30, 31]. This indicates the need to focus on RMR-TB cases so that appropriate regimens are used, even for those who are susceptible to other drugs.

Globally, the proportion of TB cases with RMR-TB varies from 0 to 2% [30, 32, 33], and the highest rates are in countries or regions with high prevalence of HIV and TB [34–36]. Nunn et al. reported a RMR of 2.6% in HIV-positive cases and 0.2% in HIV-negative cases [37], similar to the results of Sandman et al. in New York [38]. In the present study, the RMR prevalence was higher in the first survey (1998) than in the other 3 years. A possible reason for the higher RMR prevalence in 1998 is that there was not a policy in place for anti-TB medicine at that time. An increased proportion of rural to urban migrants may be another explanation for the increased prevalence, because migrants may have a higher rate of RMR [31, 39].

Our multivariable analysis indicated that previously treated patients who received treatment in designated general hospitals were less likely to have MDR-TB, indicating possible better TB management by designated hospitals than TB dispensaries. An alternative explanation may be that previously treated patients were willing to go to TB clinics in TB dispensaries when the next TB episodes happened. This topic requires further study. In our analysis of risk factors for MDR-TB among previously treated patients, those who received previous treatment for less than 6 months were less likely to develop drug resistance or MDR-TB. This could be because those treated for longer than 6 months had multiple treatment interruptions, and drug-susceptible TB patients treated for less than 6 months will have more chance of relapse than TB cases treated with standard 6 months regimen. China has long experienced inadequate TB control, and the misuse of anti-TB drugs and the development of TB-resistant strains tend to increase when treatment and management are inappropriate. The higher prevalence of MDR TB in those who received treatment for more than 6 months may also partly be because a certain proportion of patients received anti-TB treatments before 1992 when directly observed therapy (DOT) began in the province.

This study has several limitations. First, we probably underestimated the burden of drug-resistant TB because the detection rate in China is only 70% [1]. Second, this survey did not collect information on HIV status because patients with TB are not routinely tested for HIV in China; therefore, we were unable to analyze the impact of HIV prevalence on the relationship between RR-TB and MDR-TB. Finally, the weak relationship between rifampicin resistance and MDR-TB could conceivably be attributed to laboratory error [16], but we consider this to be highly unlikely because of strict compliance to the WHO guidelines for DST.

In conclusion, there is a high proportion of RMR-TB among new RR-TB cases in Zhejiang China. Thus, the strategy of using RR-TB as a proxy for MDR-TB may be unsatisfactory in this setting, especially when it is used for the new patients of tuberculosis. The management of treatment with the rapid and accurate diagnosis of MDR-TB other than only relying on RIF susceptibility testing is crucial for improving adherence and outcomes in patients with drug-resistant TB.

## Figures and Tables

**Table 1:** Resistance to anti-TB drugs among new cases and previously treated cases in 4 surveys of Zhejiang province.

	1998		2003		2008		2013		Total	
	New cases	PTC	New cases	PTC	New cases	PTC	New cases	PTC	New cases	PTC
	*n* (%)	*n* (%)	*n* (%)	*n* (%)	*n* (%)	*n* (%)	*n* (%)	*n* (%)	*n* (%)	*n* (%)
Total	806	148	832	153	842	96	853	102	3333	499
Resistance to at least one drug	119 (14.8)	87 (58.8)	220 (26.4)	80 (52.3)	224 (26.6)	48 (50.0)	192 (22.5)	48 (47.1)	755 (22.7)	263 (52.7)
Monodrug resistance										
INH	22 (2.7)	11 (7.4)	33 (4.0)	7 (4.6)	29 (3.4)	4 (4.2)	37 (4.3)	6 (5.9)	121 (3.6)	28 (5.6)
RIF	13 (1.6)	8 (5.4)	7 (0.8)	4 (2.6)	8 (1.0)	2 (2.1)	8 (0.9)	3 (2.9)	36 (1.1)	17 (3.4)
SM	2 (0.2)	1 (0.7)	98 (11.8)	13 (8.5)	117 (13.9)	6 (6.3)	75 (8.8)	5 (4.9)	292 (8.8)	25 (5.0)
EMB	29 (3.6)	7 (4.7)	8 (1.0)	1 (0.7)	3 (0.4)	0 (0.0)	7 (0.8)	2 (2.0)	47 (1.4)	10 (2.0)
RIF-resistance^*∗*^	51 (6.3)	65 (43.9)	41 (4.9)	53 (34.6)	43 (5.1)	32 (33.3)	44 (5.2)	30 (29.4)	179 (5.4)	180 (36.1)
MDR^#^	35 (4.3)	52 (35.1)	30 (3.6)	45 (29.4)	28 (3.3)	28 (29.2)	35 (4.1)	26 (25.5)	128 (3.8)	151 (30.3)
Resistance to four 1^st^ line drugs	9 (1.1)	18 (12.2)	11 (1.3)	10 (6.5)	7 (0.8)	12 (12.5)	13 (1.5)	10 (9.8)	40 (1.2)	50 (10.0)
Susceptibility	687 (85.2)	61 (41.2)	611 (73.4)	73 (47.7)	618 (73.4)	48 (50.0)	661 (77.5)	54 (52.9)	2577 (77.3)	236 (47.3)

INH: isoniazid; RIF: rifampicin; EMB: ethambutol; SM: streptomycin; PTC: previously treated cases. ^*∗*^Defined as resistant to at least RIF. ^#^Defined as resistant to at least INH and RIF.

**Table 2:** Proportion of RMR-TB among RR-TB patients from 1998 to 2013.

Year	Overall percentage of RMR-TB	RMR-TB in new RR-TB case	RMR-TB in previously treated RR-TB cases	RMR-TB in male RR-TB cases	RMR-TB in female RR-TB cases
	% (CI 95%)	% (CI 95%)	%(CI 95%)	%(CI 95%)	%(CI 95%)
1998	18.1 (11.1–25.1)	25.5 (13.5–37.5)	12.3 (4.3–20.3)	18.5 (10.1–27.0)	17.1 (4.7–29.6)
2003	11.7 (5.2–18.2)	17.1 (5.6–28.6)	7.5 (0.4–14.6)	10.4 (3.1–17.8)	14.8 (1.4–28.2)
2008	13.3 (5.6–21.0)	18.6 (7.0–30.2)	6.3 (-2.1–14.7)	14.5 (5.2–23.9)	10.0 (-3.1–23.1)
2013	14.9 (6.8–23.0)	18.2 (6.8–29.6)	10.0 (-0.7–20.7)	14.3 (4.5–24.1)	16.0 (1.6–30.4)
Total	14.8 (11.1–18.5)	20.1 (14.2–26.0)	9.4 (5.1–13.7)	14.7 (10.3–19.1)	15.0 (8.2–21.7)

RMR-TB: rifampicin mono-resistant tuberculosis; RR-TB: rifampicin resistant tuberculosis.

**Table 3:** Multivariate analysis of factors associated with drug-resistant TB among new cases and previously treated cases in 2013.

Risk factor	New cases				Previously treated cases			
	AOR (95% CI), drug-resistant TB	*P*	AOR (95% CI), MDR	*P*	AOR (95% CI), drug-resistant TB	*P*	AOR (95% CI), MDR	*P*
Age (years)		0.787		0.090		0.568		0.143
<20 yr	1		1		NA	NA	NA	NA
20–39 yr	0.94 (0.44–1.99)	0.867	NA	NA	1		1	
40–59 yr	0.86 (0.55–1.33)	0.490	0.31 (0.46–4.49)	0.141	0.66 (0.17–2.50)	0.536	0.39 (0.06–2.42)	0.311
≥60 yr	1.06 (0.69–1.64)	0.798	1.44 (0.47–4.30)	0.533	1.21 (0.38–3.86)	0.750	1.76 (0.42–7.30)	0.437

Sex								
Male	0.79 (0.55–1.14)	0.205	2.67 (0.88–8.08)	0.082	1.32 (0.46–3.81)	0.602	3.07 (0.83–11.3)	0.093
Female	1				1		1	

Occupation								
Farmer	1.10 (0.77–1.58)	0.603	1.42 (0.47–4.30)	0.538	1.50 (0.52–4.38)	0.456	2.20 (0.56–8.67)	0.259
Others^*∗*^	1				1		1	

Diabetes								
Yes	1.15 (0.67–1.97)	0.612	1.15 (0.29–4.50)	0.844	1.14 (0.28–4.61)	0.852	1.39 (0.23–8.34)	0.719
No	1				1		1	

Hepatitis B virus infection								
Yes	0.97 (0.50–1.89)	0.940	1.44 (0.65–3.16)	0.366	3.96 (0.76–20.72)	0.103	1.65 (0.72–3.77)	0.239
No	1		1		1		1	

Duration of treatment with TB drugs in the first episode of TB								
<6 months	NA	NA	NA	NA	0.40 (0.16–0.97)	**0.043** ^**†**^	0.24 (0.07–0.81)	**0.022** ^**†**^
≥6 months					1		1	

Medical facility providing first TB treatment								
TB clinic system					1		1	
General hospital or other TB hospital	NA	NA	NA	NA	0.51 (0.18–1.47)	0.21197	0.08 (0.01–0.72)	**0.025** ^**†**^

TB: tuberculosis, AOR: adjusted odds ratio, MDR-TB: multidrug-resistant tuberculosis, CI: confidence interval; NA: not applicable. ^†^: *P* < 0.05. Other occupation indicates the following: Worker, Migrant labourer, Students, Retiree, Catering staff, etc.

## Data Availability

The datasets generated and analyzed from the current study are not publicly available at this time as further analyses are ongoing but are available from the corresponding author upon reasonable request.
